# Impacts of Climate Changes on Geographic Distribution of *Primula filchnerae*, an Endangered Herb in China

**DOI:** 10.3390/plants12203561

**Published:** 2023-10-13

**Authors:** Xin Jiang, Wan-Jing Liu, Yan-Zhao Zhu, Yu-Ting Cao, Xiu-Min Yang, Yao Geng, Fu-Jiao Zhang, Rui-Qi Sun, Rui-Wen Jia, Chun-Li Yan, Yang-Yan Zhang, Zhong-Hu Li

**Affiliations:** Key Laboratory of Resource Biology and Biotechnology in Western China, Ministry of Education, College of Life Sciences, Northwest University, Xi’an 710069, China; jiangxin@stumail.nwu.edu.cn (X.J.); vankinglau@163.com (W.-J.L.); 18855078692@139.com (Y.-T.C.); 17865561551@163.com (X.-M.Y.); ygeng1119@163.com (Y.G.); zhangfj08@126.com (F.-J.Z.); srq981124@163.com (R.-Q.S.);

**Keywords:** species distribution, MaxEnt model, climate response, environmental factors, conservation strategies

## Abstract

*Primula filchnerae*, an endangered plant endemic to China, has drawn people’s attention in recent years due to its ornamental value in flower. It was rarely recorded since being described in 1902, but it was rediscovered in 2009 and is now known from a limited number of sites located in Hubei and Shaanxi Provinces. Since the species is still poorly known, a number of unanswered questions arise related to it: How has *P. filchnerae* responded to past climate change and how might it respond in the future? Why was *P. filchmerae* so rarely collected during the past century? We assembled geographic coordinates for *P. filchnerae* through the field surveys and website searches, and then used a maximum entropy model (MaxEnt) to simulate its potential suitable distribution in six periods with varied carbon emission levels by combining bioclimatic and environmental factors. MaxEnt showed that Min Temperature of the Coldest Month (bio6) and Precipitation of the Coldest Quarter (bio19) affected *P. filchnerae*’s distribution most, with an aggregate contribution >60% and suitable ranges above −5 °C and below 40 mm, respectively. We also analyzed potential habitat distribution in various periods with differing impacts of climate change compared to today’s suitable habitats, and in most cases, Shaanxi and Sichuan remained the most stable areas and with possible expansion to the north under various carbon emission scenarios, but the 2050s SSP5-8.5 scenario may be an exception. Moreover, we used MaxEnt to evaluate population shifts, with various scenarios indicating that geometric center would be concentrated in Sichuan Province in China. Finally, conservation strategies are suggested, including the creation of protected areas, long-term monitoring, raising public awareness of plant conservation, situ conservation measures, assisted migration, and species introduction. This study demonstrates how *P. filchnerae* may have adapted to changes in different periods and provides a scientific basis for germplasm conservation and management.

## 1. Introduction

Over the past century, the global temperature has risen by close to 1 °C, and it has increased rapidly in the past 30 years. The climate is one of the most essential environmental factors that affects the distribution and geographic ranges of species [1]. Human activities also play a vital role in the geographic distribution of species [2]. In recent years, more and more attention has been paid to how plants adapt to climate change, and the study of the species spatial patterns has become a hot issue [3]. As global warming increases, the spatial distribution patterns of plants are expected to move to higher latitude [4]. Future climate change will alter the land surface temperature, precipitation pattern, and affect the geographical distribution patterns of plants under the condition of global warming. With climate changes, the distribution range of plants will alter in response to the climate oscillations, yet projections for various species show differing changes, in which some species’ distribution would expand but some would not. For instance, the suitable habitat range of Chinese *Ziziphus jujuba*, a deciduous shrub with economic value, would increase with climate warming [5], while the high suitable areas of *Magnolia wufengensis* under the RCP6.0 scenario is 39.14% lower than the current total of high suitability areas [2]. The global climate change has not only resulted in shifts in the habitats of various species, but also induced the extinction of some species [6,7]. So, it is necessary for us to focus on the response of species to climate change over the past years and in the future because the responses of plants to the climate change will not only help us to understand the historical causes of species formation and changes in the geographical distribution but also in formulating scientific management strategies [5].

It has been widely accepted that it is possible to understand changes in species adaptability and predict potential geographical distribution by simulating and predicting the geographical distribution of species under changing climate conditions. [8]. Species distribution models have been widely used to study the potential geographic distribution of species under various climate conditions [9]. Species distribution models (SDMs), also called ‘habitat’ models, can assess the distribution of a given species simply based on presence data and various environmental parameters. SDMs can also be used to estimate the spatial variation of species richness, look for the sources of changes, and determine possible migration directions [10]. As computer technology and geographic information systems (GIS) develop, numerous SDMs and SDM software packages have been explored, such as CLIMEX, BIOCLIM, GAPR, and MAXENT [11,12]. Each model has pros and cons due to their different principles and algorithms, and the performance of each model becomes unstable if the input data are changed [13]. Among them, the MaxEnt model, derived from statistical mechanics based on maximum entropy [14], relies on presence-only data and obtains better simulation results even with small samples [15,16]. The MaxEnt model has been considered as one of the most reliable SDMs by numerous researchers [17,18,19]. For instance, some researchers explored how rhesus macaques adapted to climate change by integrating ecological and genetic methods, applying species distribution models (SDMs) [20]. Other researchers integrated the habitat and saponin content of *Panax notoginseng* to assess its potential distribution by MaxEnt model [21]. The potential geographic distribution of endangered species has been the focus of attention in numerous studies, such as the effects of climate change on the spatial distribution of the threatened species *Rhododendron* in the Qinling-Daba mountains of central China [22]. Another example is the simulation of potential suitable distribution of the endangered medicinal of *Paeonia rocii* under climate change scenarios via maxent modelling [23].

*Primula* is one of the largest genera of Primulaceae including approximately 500 species, which are mainly distributed in the temperate and arctic regions of the northern hemisphere, with about 300 species native to China [24]. Some species, including *P. malacoides, P. vulgaris*, and *P. veris* ‘Sunset Shades’, are widely cultivated as garden plants and well-known for their early flowering period. *Primula filchnerae* is endemic to central China [25] and was evaluated as an Endangered species according to the IUCN (The International Union for Conservation of Nature) Red List Categories and Criteria [26]. It was first discovered at the beginning of the 20th century, but then disappeared from the wild for about 100 years. Its wild populations were rediscovered in 2009 in the Province of Hubei [27] and in 2015 in the province of Shaanxi [28,29]. *Primula filchnerae* is a biennial herb, with multiple clusters of oval leaves and umbels [25], feathery leaves, bell-shaped fruits, and brightly colored flowers, growing at an altitude of between 200 m and 900 m above sea level, specifically along roadsides or among the rocks. The most common companion plants from our field surveys were *Anemone vitifolia*, *Glycine soja, Oplismenus undulatifolius*, and *Zanthoxylum armatum.* In the past several years, the Xian Botanic Garden in Shaanxi Province expanded the population of *P. filchnerae* by collecting their seeds from the wild, translocating them to the garden, and returning cultivated seedings to wild sites. As an endangered species in China with a long period when it had been unnoticed, the appearance of *P. filchnerae* amazed numerous researchers who could not help keep asking why *P. filchnerae* had been missing for such a long time, how *P. filchnerae* has responded to past climate change and how it might respond in the future. Studying the potential geographic distribution of *P. filchnerae* under climate change is helpful to better understand the adaptation of *P. filchnerae* to climate change and take targeted measures for its further protection.

In this study, we collected distribution data through field surveys and website searches during the past two years, which were employed to analyze by ENMeval to reduce redundancy. MaxEnt model was employed to perform the relevant analysis based on the occurrence records. Our objectives this study were to: (1) analyze the contributions of environmental factors and explore the essential climatic factors limiting *P. filchnerae*’s distribution; (2) simulate and predict the potential spatial distributions and suitable habitats of *P. filchnerae* in different periods with varied emission level of carbon dioxide; (3) study the expansion and contraction in future compared with current distribution areas; and (4) model the migration route of population in different periods to determine the ideal areas for species conservation.

## 2. Results

### 2.1. Analysis of the Accuracy of the MaxEnt Model

**Figure 1:**
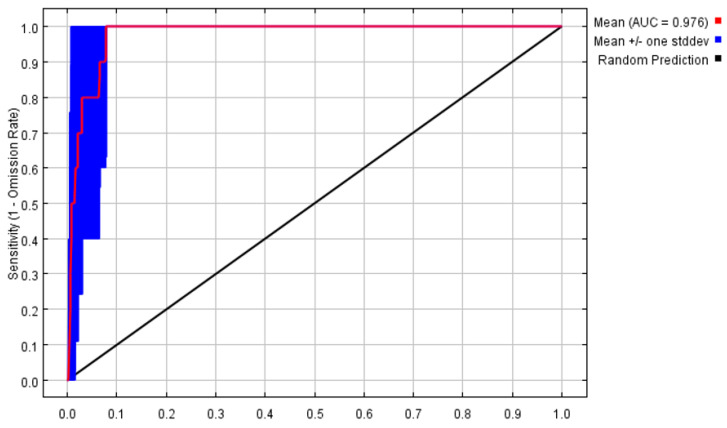
Receiver operator characteristic curve tests the accuracy of MaxEnt model.

The MaxEnt model was used to predict the potentially suitable areas, and the results of AUC values are presented in (Figure 1). Its average test data of 10 times were 0.976 and the performance of the MaxEnt model was ‘excellent’, which could well predict the geographic distributions of *P. filchnerae*.

### 2.2. Evaluation of Environmental Variables and Analysis of Response Curve

**Figure 2:**
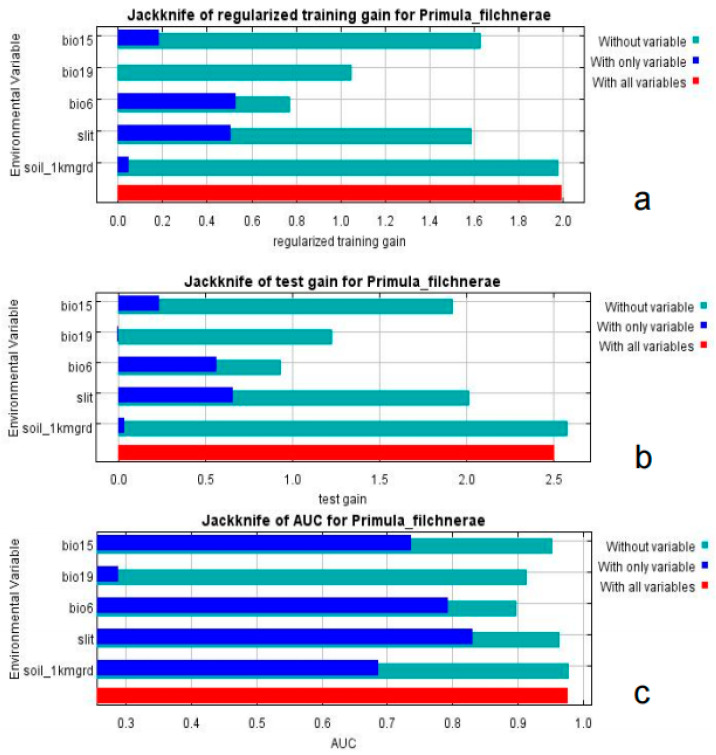
Jackknife test of importance of environmental variables. (**a**) Regularized training gain; (**b**) test gain; (**c**) AUC value.

**Figure 3:**
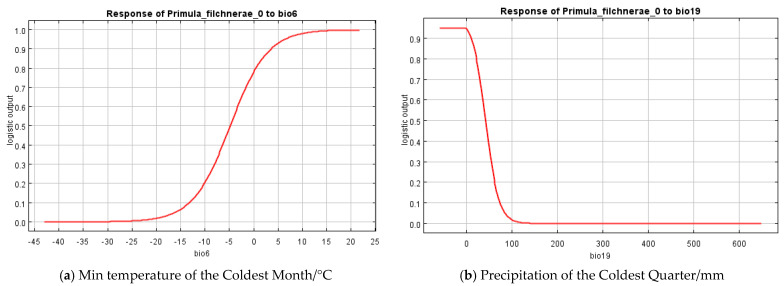
Response curves of the most important variables in the ecological niche model for *P. filchnerae*. (**a**) Min temperature of the coldest month (bio6); (**b**) Precipitation of the coldest quarter (bio19).

Based on the percent contribution of the results of the MaxEnt model, Min Temperature of the Coldest Month (bio6), Precipitation Seasonality (bio15), Precipitation of the Coldest Quarter (bio19), soil_1kmgrd, and silt (Figure 2) were the most influential environmental variables, in which Min Temperature of the Coldest Month (bio6) and Precipitation of the Coldest Quarter (bio19)’s percent contribution was more than 65%. Single-factor response curves were also draw by logistic regression in the MaxEnt model. The Min Temperature of the Coldest Month was above −5 °C (Figure 3) and the precipitation of the coldest quarter was below 40 mm (Figure 3) with a probability of presence >0.5.

### 2.3. Distribution Areas Predicted in China

#### 2.3.1. Suitable Areas in the Past

**Figure 4:**
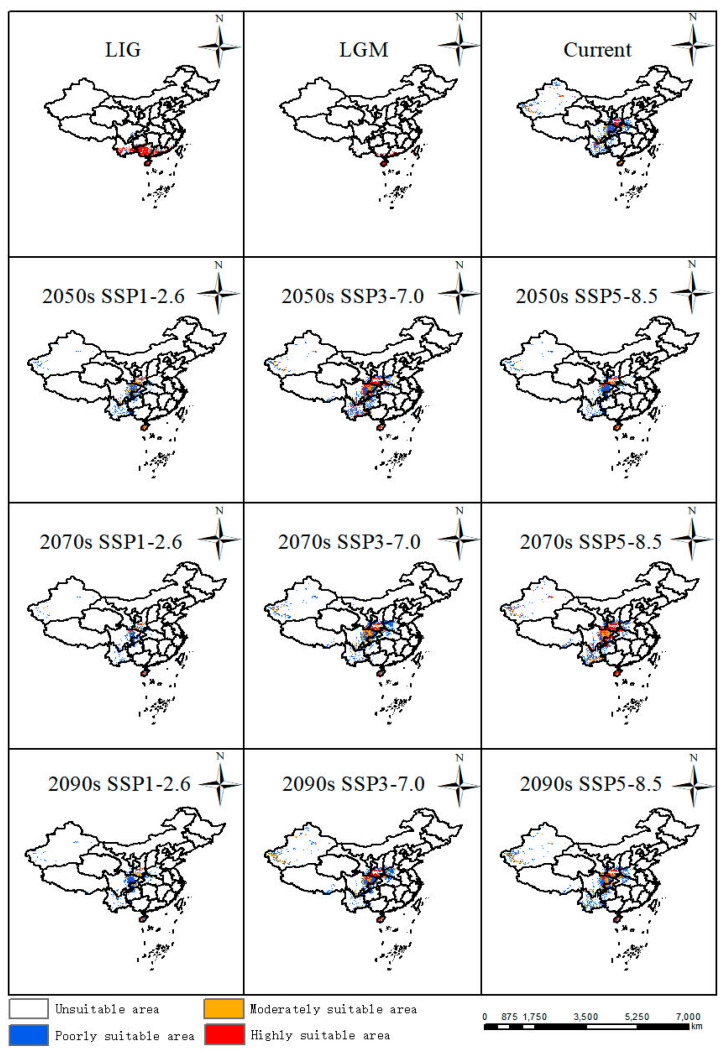
Potentially suitable areas of *P. filchnerae* in China under different climate scenarios in various periods.

In comparison with the current distribution, suitable areas in the LIG (about 130 Ka BP, Kilion-anniversary Before Present) and LGM (about 21 Ka BP, Kilion-anniversary Before Present) showed a trend towards the north of China. In LIG, the largest areas were highly suitable areas mainly distributed in Yunnan Province, Hainan Province, Guangdong Province, and Guangxi Zhuang Autonomous Region in China (Figure 4). In LGM, its suitable habitats decreased a lot, especially in Yunnan Province and Guangxi Zhuang Autonomous Region, while suitable areas in Hainan Province remain stable from LIG to LGM (Figure 4).

#### 2.3.2. Suitable Areas at the Present Time

The results showed that the suitable area was 70.86 km^2^ [Table 1], which accounted for 7.38% of China’s land area, mainly located in southern and central China (Figure 4). Among the highly suitable areas in China, Shaanxi covered the largest areas, which was in accordance with current occurrence records. Suitable areas were also distributed in Sichuan Province, Yunnan Province, Hainan Province, and Xinjiang Uygur Autonomous Region.

#### 2.3.3. Suitable Areas in the Future

Based on future habitat predictions, highly and moderately suitable areas displayed the largest expansions in the 2050s under the SSP3-7.0 scenario compared with other scenarios in the 2050s, while the suitable habitats showed an increasing trend from SSP1-2.6 to SSP5-8.5 in the 2070s and part of the suitable habitats were also distributed in Hubei Province, Chongqing City, and Guizhou Province. Similarly to the change patterns in the 2050s, suitable areas displayed the largest expansion in the 2090s under the SSP3-7.0 scenario (Figure 4).

### 2.4. Possible Climate Effect on the Habitat of P. filchnerae

**Figure 5:**
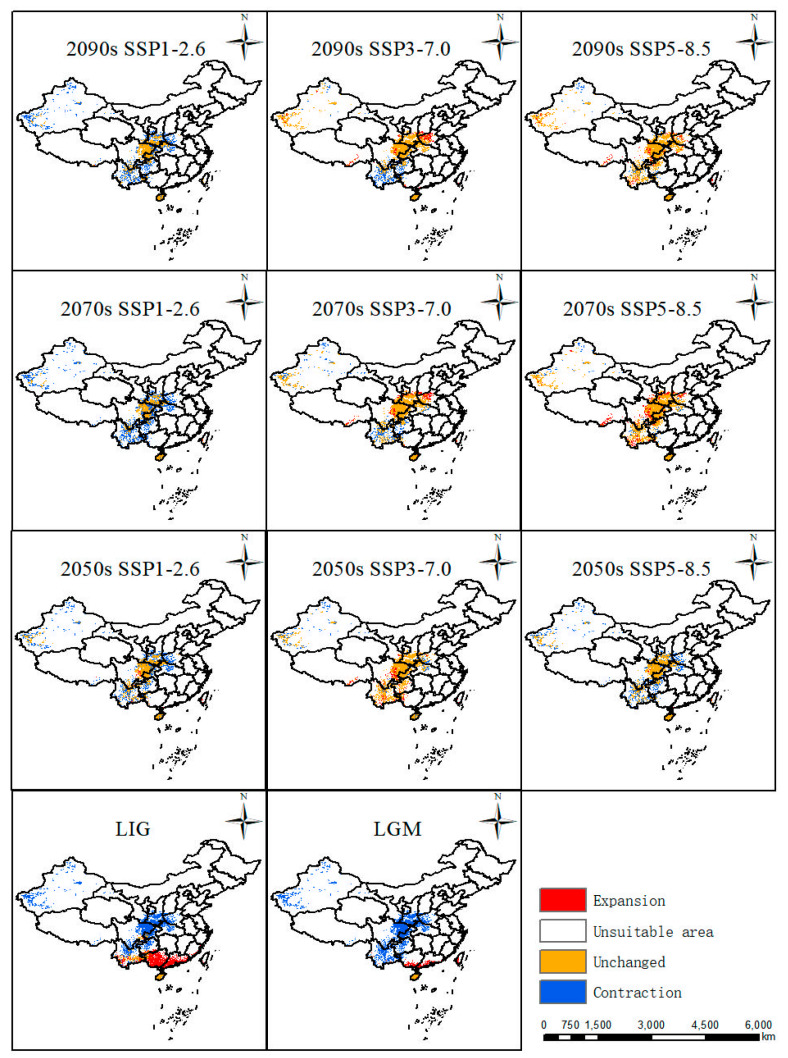
Changes in suitable areas of *P. filchnerae* relative to the current climate change scenarios.

The predicted distribution patterns in various periods were compared with the current distribution (Figure 5), and its distribution in different periods under various scenarios witnessed different trends. From last interglacial period to the current, Guangxi and Guangdong experienced the largest increases in suitable habitat compared with changes in other periods. From the last glacial maximum to the present, it can be seen that Guangxi and Guangdong also experienced increases in suitable areas in their southern areas. From the 2050s, 2070s, and 2090s under three scenarios, they displayed almost the same distribution patterns. Under the SSP1-2.6 scenario, the most stable areas were Shaanxi Province and Sichuan Province, with the largest contraction areas in the Xinjiang Uygur Autonomous Region, Yunnan Province, Guizhou Province, Henan Province, and Hubei Province. Under the scenario SSP3-7.0 in the 2050s, the suitable distribution pattern displayed the most stable distribution compared with the 2070s and 2090s, and the major contraction areas were in Yunnan Province and Guizhou Province. Meanwhile, under the scenario SSP5-8.5, the distribution pattern showed a similar pattern in the 2090s and 2070s with a minor contraction in Xinjiang. Nevertheless, the 2050s covered more contraction areas in Xinjiang, Yunnan, Guizhou, Henan, and Hubei Provinces.

### 2.5. The Migratory Route of the Geometric Center of P. filchnerae

**Figure 6:**
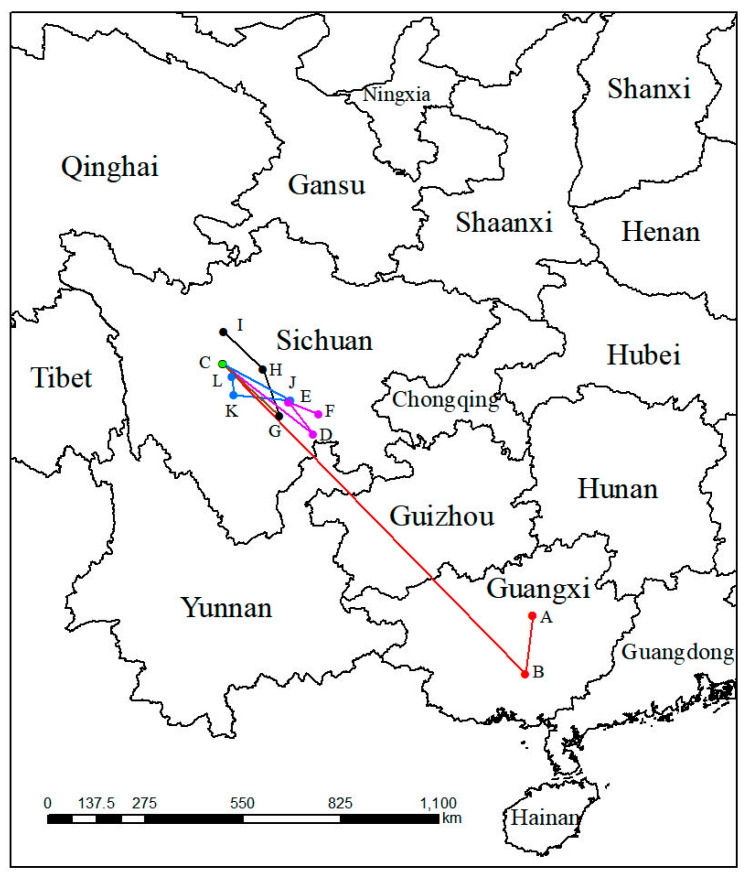
Migration of the center of suitable habitat for *P. filchnerae* since the last interglacial period. The letters refer to (A) LIG, (B) LGM, (C) Current, (D) 2050s-SSP1-2.6, (E) 2070s-SSP1-2.6, (F) 2090s-SSP1-2.6, (G) 2050s-SSP3-7.0, (H) 2070s-SSP3-7.0, (I) 2090s-SSP3-7.0, (J) 2050s-SSP5-8.5, (K) 2070s-SSP5-8.5, and (L) 2090s-SSP5-8.5.

In the past, the geometric centers (point A and point B) of suitable areas for both of LIG and LGM were in the Guangxi Zhuang Autonomous Region (Figure 6). From the past to the present, the geometric center moved toward the southwest about 167.63 km, then kept moving toward the northwest 1217.62 km to the Sichuan Province, the geometric center of the suitable distribution in the modern period. Under SSP1-2.6 in various periods, the geometric center would move toward the southeast about 321.18 km at an average hypothetical speed of 6.42 km/year at first, then move to the northwest and southwest 111.36 km at an average hypothetical speed of 5.56 km/year and 89.21 km at an average hypothetical speed of 4.46 km/year, respectively. Nevertheless, the geometric center of migratory route of the suitable distribution under the SSP3-7.0 compared with the route under the SSP1-2.6, existed differences from the 2070s to the 2090s, when the route was toward the northwest of China. Under SSP5-8.5, unlike the migratory route under SSP3-7.0 from the 2050s to the 2070s, its direction was toward the northwest with a distance of 160.17 km (Table 2).

## 3. Discussion

Results from MaxEnt showed that Min Temperature of the Coldest Month (bio6), Precipitation Seasonality (coefficient of variation) (bio15), Precipitation of the Coldest Quarter (bio19), silt, and soil_1kmgrd were the dominant factors affecting the suitable distribution for *P. filchnerae*. In terms of the percentage contribution of the variables, the total contribution of Min Temperature of the Coldest Month (bio6) and Precipitation of the Coldest Quarter (bio19) was more than 65%, while if we take the Jackknife test into consideration, Min Temperature of the Coldest Month (bio6) and silt were the most dominant factors affecting the distribution of *P. filchnerae*. In general, Min Temperature of the Coldest Month (bio6) (>−5 °C), Precipitation of the Coldest Quarter (bio19) (<40 mm), and silt were highly significant for the distribution of *P. filchnerae*. It is clear that climate factors are crucial determinants of species distributions at different scales [30]. Min Temperature of the Coldest Month (bio6) determines whether *P. filchnerae* could survive and this was consistent with previous similar research [31]. Researchers also found the precipitation of warmest and coldest quarters (bio18 and bio19) to be the highest weight cofactors for projecting the future potential distribution of high-value medical plants in Nepal [32]. *Primula filchnerae* has a flowering period from February to April, and its growth is closely related to temperature, especially the Min Temperature of the Coldest Month that determines whether *P. filchnerae* can survive the coldest weather in the winter. Moreover, our results also indicated that precipitation was also an important factor that restricted *P. filchnerae*’s suitable habitats. Water was reported to play an indispensable role in root growth, release of seed dormancy [33,34,35], and promotion of germination, which is consistent with our observations that seeds of *P. filchnerae*, when held in Petri dishes and covered with water, would germinate within several days.

Based on the results of the MaxEnt model, the suitable distribution habitats were mainly in Shaanxi Province, Sichuan Province, and Yunnan Province, among which Shaanxi Province was the sampling field of this study, or to be more exact, the central of the Qinglin Mountains and its adjacent areas, and was also the largest of the predicted areas from this model, a finding supported by many previous studies that predicted ranges that were consistent with the actual distribution of certain species [36,37]. The Qinling Mountains are home to many endangered species due to their unique geological environment and climate, among which local trees, and herbs, such as *Notopterygium oviforme* [38], *Abies chensiensis* [39], were usually employed to predict and simulate its geographic distribution in future under the impact of climate change. During the last interglacial period and the last glacial maximum period, compared with the current suitable distribution, the suitable distributions were mainly distributed in Yunnan Province, Guangxi Province, Hainan Province, and Guangxi Zhuang Autonomous Region, which indicated an obvious trend for a real reduction from LIG to LGM, more precisely from the north to the south. And this may be due to the climate during the last interglacial period being warm and humid, while the last glacial maximum was harsher, resulting in suitable area loss from LIG to LGM [40,41,42]. This was in line with the temperature profile of China, which tended to be warmer in the south than in the north, so as the climate changed, *P. filchnerae* may have migrated to warmer areas, such as Hainan Province during the LGM period. Under future climate change, Shaanxi Province and Sichuan Province should remain the most stable distribution areas; nevertheless, the northern part of the modern suitable habitat shows varying degrees of area increase, and the southern part of the modern suitable habitat is projected to shrink [5]. The contraction areas are greater than the increased areas in the future under climate change, except the scenarios SSP5-8.5 in the 2070s and 2090s, which was supported by a similar plant, *Larix potaninii* Batalin, whose suitable habitats would be reduced by 24.66 × 10^4^ km^2^ (9.59% of QTP) under the 2081-2100 SSP5-8.5 scenario [43]. Under the SSP1-2.6 scenario, the loss of suitable areas was larger than other scenarios in various periods, among which Yunnan Province, Guizhou Province, Hubei Province, and Henan Province were the main decreased areas. Under the SSP3-7.0 scenario, the decreased suitable areas are associated positively with time, while under the scenario in the 2050s (contraction > expansion), 2070s (contraction < expansion), and 2090s (contraction < expansion), the situations were a little different. Suitable habitats increased in different periods and scenarios, but did not cross the boundary of central Shaanxi Province, presumably because the Qinling Mountains act as a barrier to northward migration. Furthermore, climate change in the future would result in the geographical migration mainly caused by increased temperature and decreased precipitation [44]. From LIG to LGM, *P. filchnerae* had the longest migration distance, 1217.62 km, to the north, and this may be related to the dramatic climate change from LIG to LGM compared with climate change between other periods. Moreover, under these varying future scenarios, the migration routes of the center did not always keep the same direction, though overall, the center of suitable habitats kept migrating toward to the northwest of China. These results are consistent with other studies reporting that climate change, especially climate warming, resulted in species migration toward higher latitudes [45,46].

Climate change is one of the most significant drivers of plant distribution and is equally important in limiting plant dispersal [47], which determines whether plants can survive or reproduce in another location. Human activity is also an important factor in preventing the spread of plants [48], leading to ranges that are reduced and fragmented. In addition, other ecological variables also play an important role in the distribution and dispersal of species, such as specific soil composition, geographical barriers, and competition between native plants and newly invaded plants. For example, the ecological requirements of *Primula* sect. *Auricula* species vary in soil_1kmgrd (soil type), and most of them require basic soils with limestone or acid soils with granite, schist, or similar materials [49], which could possibly explain the importance of soil types for *Primula* species given habitats locations, but there are no detailed reports about soil composition requirements for *P. filchnerae*. Some of these environmental factors were not studied in this study due to limited information and methods.

However, some specific events can also affect the dispersal of plants, such as the flight of a dandelion fruit or animals consuming seeds, or the extreme events of seed release after burning or explosive ejection [50]. It is important to study the mechanisms of seed dispersal and link them with the environments, because they are essential to understand the population dynamics and distribution. Growing recognition of the importance of long-distance dispersal (LDD) of plant seeds for various ecological and evolutionary processes has led to an upsurge in research into the mechanisms underlying LDD [51]. Long-distance dispersal (LDD) events were typically rare, yet play a major role in determining large-scale processes such as population spread, the flow of individuals between populations, the colonization of unoccupied habitats, and the assembly of local communities from the metacommunity. Two types of LDD have been defined: one is a passive mechanism, including wind dispersal, animal dispersal (migratory animals), and explosive dispersal, and another was an informed mechanism. The informed mechanism has been largely referred to as animal dispersal whereby individuals can acquire information about their environment to make decisions about their movements [50]. However, there were also many cases that plants can exert control over dispersal by altering their development or responding to environmental conditions. For instance, the brassica *Aethionema arabicum* forms dehiscent fruits that open to release seeds directly into the nearby proximity, as well as indehiscent winged fruits via developmental plasticity that carry seeds further by wind. In addition, a greater proportion of the winged indehiscent fruits are produced that may disperse further, when they are at a higher altitude and a harsher, less predictable environment [52,53]. Based on our observations, fruits of *P. filchenerae* are bell-shaped, without an explosive dispersal mechanism, and are more likely to be transported by birds and mammals, and these migratory birds and mammals may have a considerably higher velocity than equivalent nonmigratory animals. And migratory animals were more likely to transport seeds across dispersal barriers, such as mountains and rivers. Moreover, the mature seeds were so small and light that wind dispersal could help. Some researchers found that seeds dispersed by wind had higher LDD in more open landscapes by mechanistic models [54]. Given the limited information on biological traits and the limitations of our knowledge, comprehensive research about the mechanism of *P. filchnerae* dispersal will be conducted in the future. Of course, the future of seed dispersal depends on the continuation of reliable seed production. Since many *Primula* species are self-incompatible, such as *P*. *forbesii, P. vulgaris, and P. veris* [55,56,57], studies to identify effective pollinators of *P. filchnerae*, their current distributions, and their ability to migrate should also be investigated.

Protecting plant diversity is of utmost importance for mitigating the impacts of climate change, as diverse plant communities contribute to carbon sequestration, soil stabilization, and regulation of local climates. *Primula filchenerae* has been classified as an endangered species [26] with highly ornamental value, so it is necessary to take conservation measures to protect this species. Based on our field surveys, we found that human activities (especially road construction), small populations, and fragmented habitats have significantly reduced the wild population of *P. filchenerae* [58,59]. However, specific management and conservation strategies were still lacking. To ensure the long-term conservation of *P. filchenerae*, in situ conservation measures can be implemented in the current suitable habitats of *P. filchenerae* [23]. Adaptive measures included improving the effectiveness of protected areas, long-term monitoring, and raising public awareness of plant conservation [60,61]. In addition, the predicted suitable habitats under future climate conditions should be considered as priority areas for assisted migration and species introduction [62]. Moreover, ex situ conservation measures, including botanical gardens and arboreta, can be used to conserve germplasm resources and expand the population. For example, the Xian Botanical Garden in Shaanxi Province has successfully cultivated *P. filchenerae* and conducted field regression experiments to expand its wild population [63].

## 4. Materials and Methods

### 4.1. Occurrence Data and Its Distribution

**Figure 7:**
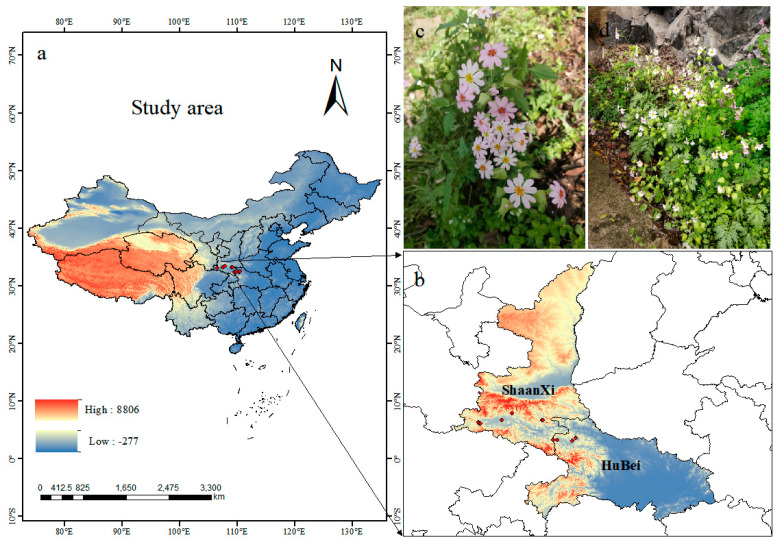
(**a**,**b**) Spatial distribution of *P. filchnerae* occurrences recorded in China; (**c**,**d**) photos of *P. filchnerae* in field surveys.

Fourteen location records of *P. filchnerae* were assembled from (1) field surveys of the natural populations of *P. filchnerae* in Shaanxi and Hubei in 2023, (2) the Global Biodiversity Information Facility (GBIF, http://v5.cvh.org.cn/ (accessed on 1 September 2023)), (3) the Chinese Virtual Herbarium (http://www.cvh.ac.cn (accessed on 1 September 2023)), and (4) a search of the published literature. ENMeval program was used to reduce the sampling deviation impact by deleting redundant data within 5 km^2^ on the ground. Consequently, 11 records were selected for display and further analysis by ArcGIS 10.8 (ESRI, Redlands, CA, USA) (Figure 7).

### 4.2. Variable Selection

**Figure 8:**
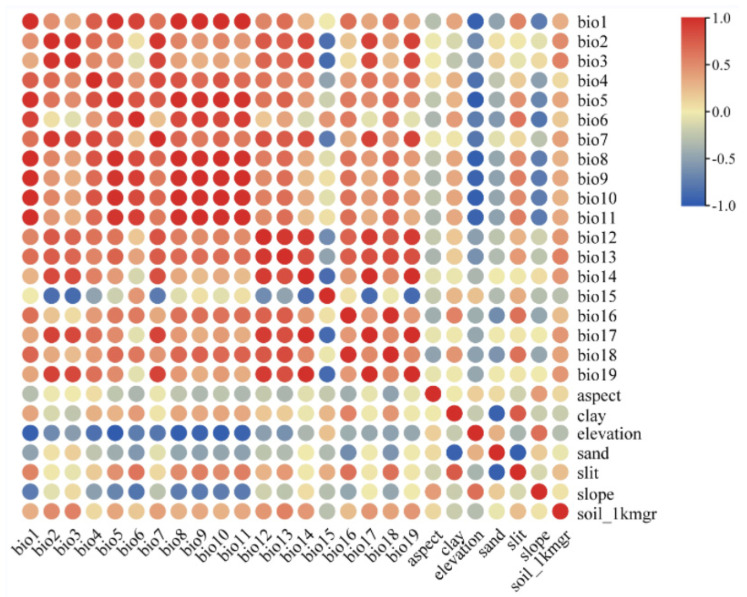
Correlation analysis of various factors. The correlation coefficient values rise between two variables with the color becoming darker. Red means high correlation, and blue represents low correlations.

A total of 26 environmental factors (Table 1) with 2.5 arc-minute (~5 km^2^) resolution were initially used to construct the MaxEnt model, among which 19 bioclimatic variables and 3 topographical variables were downloaded from the model of BCC-CSM2-MR in the World Climate Database (WorldClaim, http://worldclim.org/ (accessed on 1 September 2023)) released by the Coupled Model Intercomparison Project Phase (CMIP6), including the last interglacial period, the last glacial maximum period, and four additional periods: Current (1970–2000), the future 2050s (2040–2060), the future 2070s (2060–2080), and the future 2090s (2080–2100), which include the four shared socioeconomic paths SSP1-2.6, SSP2-4.5 (exception), SSP3-7.0, and SSP5-8.5. They are employed to describe various socioeconomic paths in order to predict and understand the impact of climate change under different socio-economic conditions. They were used to construct MaxEnt model to predict *P. filchnerae* distribution in different periods and calculate its expansion and reduction under different climate change scenarios. Four environmental variables, clay, sand, silt, and soil_1kmgrd (representing various soil types), were obtained from the Institute of Geographic Sciences and Natural Resources Research, Chinese Academy of Sciences (http://www.resdc.cn (accessed on 1 September 2023)). In consideration of correlations between environmental variables, the contribution of environmental factors from MaxEnt and Spearman correlation analysis (Figure 8) were combined to select environmental variables. Then, pairs of variables that were correlated above |0.8| were singled out with the variable showing the lower contribution omitted. Finally, five variables (bold variables in Table 3) were used to establish the model. Environmental factors were set as continuous variables and the jackknife method employed to evaluate the importance of environmental variables. Response curves were generated to predict suitable conditions for species, with the test data set to 25%, and the model was run 1000 times and repeated 10 times. Other parameters were set to system default values.

### 4.3. Analysis of MaxEnt Model Performance

The receiver operating characteristic (ROC) curve was used to evaluate model performance by determining the area under the ROC curve (AUC) because the AUC is less affected by sample and threshold [64,65,66].

AUC values range between 0 and 1, and the higher the value, the more accurate the model prediction results [67]. The evaluation criteria are as follows: values above 0.9, which means that the model is excellent; values between 0.8 and 0.9, which indicates that the model is good; values between 0.7 and 0.8, which shows that the model is moderate; values under 0.7, which means that the model is poor. The importance of variables on the distribution of *P. filchnerae* was measured via the ‘Jackknife test’, and the impact of environmental factors on the distribution of *P. filchnerae* was analyzed using response curves [68].

### 4.4. Division of Suitable Habitat

The output results of MaxEnt were selected as the prediction result of the study in different periods, and then they were reclassified using the Reclass tool in ArcGIS 10.8, after they were converted to raster file format from ASC file output in ArcGIS 10.8 [30]. The prediction areas of *P. filchnerae* were divided into four levels based on the probability (P) of species’ presence: highly suitable habitat (>0.7), moderate suitable habitat (0.5–0.7), low suitable habitat (0.3–0.5), and unsuitable habitat (0–0.3).

### 4.5. Analysis of Area and Distribution Center Route

Areas with different levels of suitability were calculated through spatial analysis module in the ArcGIS, and the changes in area in different periods were compared with the current suitable distribution. In addition, we also employed SDMtool to analyze the centroids of suitable habitats at different times. The migratory population route was drawn to illustrate the temporal and spatial evolution of *P. filchnerae* from the past to the future in ArcGIS.

## 5. Conclusions

In this study, we used MaxEnt to model the distribution of *P. filchnerae* since the last interglacial period. The results showed that Min Temperature of the Coldest Month (bio6), Precipitation of the Coldest Quarter (bio19), and silt played a decisive role in the distribution of *P. filchnerae* over time. The results of this study indicated that Central Qinling mountains and Southwestern Sichuan were the main suitable distribution areas, which is consistent with extant occurrence records. In addition, based on a shift in geometric centers, suitable habitats were predicted to move toward the northwest of China, coalescing in Sichuan Province over time. Under future climate scenarios (2070s, 2090s under the scenarios of SSP3-7.0, SSP5-8.5), Shaanxi and Sichuan will remain the most stable suitable areas, the northern part of modern suitable habitat will expand, while the southern part will shrink. Based on the current suitable habitats, adaptive measures, such as the establishment of protected areas, long-term monitoring, and raising public awareness of plant conservation can be implemented. Ex situ conservation measures at botanical gardens and arboreta can be undertaken based on projections of suitable distribution. Overall, this study provides a scientific basis for further survey into protection against climate change in the future.

## Figures and Tables

**Table 1:** Suitable area for *P. filchnerae* under different climate change scenarios (10^4^ km^2^).

Period	Total Suitable Area	Unsuitable Area	Poorly Suitable Area	Moderately Suitable Area	Highly Suitable Area
LIG	45.38	914.62	7.00	6.89	31.48
LGM	12.73	947.27	1.85	1.8	9.09
Current	70.86	889.14	44.33	17.86	8.68
2050s (SSP1-2.6)	39.35	920.65	25.58	10.62	3.16
2050s (SSP3-7.0)	68.41	891.59	37.09	16.24	15.08
2050s (SSP5-8.5)	45.04	914.96	28.57	11.23	5.24
2070s (SSP1-2.6)	26.25	933.75	17.66	6.85	1.73
2070s (SSP3-7.0)	61.26	898.74	37.44	15.51	8.31
2070s (SSP5-8.5)	79.14	880.86	36.92	21.46	20.76
2090s (SSP1-2.6)	33.78	926.22	22.38	8.81	2.59
2090s (SSP3-7.0)	62.30	897.70	37.82	15.40	9.09
2090s (SSP5-8.5)	71.86	888.14	43.38	19.98	8.50

**Table 2:** Geometric center of the *P. filchnerae* and its migration distance and speed between different climate scenarios.

Period	Longitude	Latitude	Migration Direction	Distance (km)	Speed (km/Year)
LIG	109.366543	24.264458	LIG-LGM	167.635	-
LGM	109.162949	22.769528	LGM-Current	1217.623	-
Current	101.509978	30.613346	Current-2050ssp126	321.184	6.42
2050s SSP1-2.6	103.799766	28.849	2050ssp126-2070ssp126	111.367	5.56
2050s SSP3-7.0	102.93946	29.294675	2070ssp126-2090ssp126	89.213	4.46
2050s SSP5-8.5	103.220577	29.693136	Current-2050ssp370	216.089	4.32
2070s SSP1-2.6	103.187339	29.642448	2050ssp370-2070ssp370	138.635	6.93
2070s SSP3-7.0	102.530234	30.473384	2070ssp370-2090ssp370	153.850	7.69
2070s SSP5-8.5	101.785324	29.828095	Current-2050ssp585	215.821	4.31
2090s SSP1-2.6	103.93796	29.3574	2050ssp585-2070ssp585	160.174	8.00
2090s SSP3-7.0	101.542691	31.443977	2070ssp585-2090ssp585	53.301	2.66
2090s SSP5-8.5	101.748686	30.306405			

**Table 3:** Twenty-six environmental variables were used in this study. The variables in bold were ultimately selected to build the model to predict the potentially suitable areas for *P. filchnerae*.

Category	Variable	Description	Unit
Climate	bio1	Annual Mean Temperature	°C
	bio2	Mean Diurnal Range (mean of monthly (max temp − min temp))	°C
	bio3	Isothermality (bio2/bio7) (×100)	/
	bio4	Temperature Seasonality (standard deviation × 100)	/
	bio5	Max Temperature of the Warmest Month	°C
	**bio6**	Min Temperature of the Coldest Month	°C
	bio7	Temperature Annual Range (bio5–bio6)	°C
	bio8	Mean Temperature of the Wettest Quarter	°C
	bio9	Mean Temperature of the Driest Quarter	°C
	bio10	Mean Temperature of the Warmest Quarter	°C
	bio11	Mean Temperature of the Coldest Quarter	°C
	bio12	Annual Precipitation	mm
	bio13	Precipitation of the Wettest Month	mm
	bio14	Precipitation of the Driest Month	mm
	**bio15**	Precipitation Seasonality (coefficient of variation)	/
	bio16	Precipitation of the Wettest Quarter	mm
	bio17	Precipitation of the Driest Quarter	mm
	bio18	Precipitation of the Warmest Quarter	mm
	**bio19**	Precipitation of the Coldest Quarter	mm
Topography	Elevation		m
	Slope		°
	Aspect		rad
Soil	Clay		%
	Sand		%
	**Silt**		%
	**Soil_1kmgrd**	Soil types	/

## Data Availability

Data available on request from the authors.

## Abbreviations

MaxEnt, a maximum entropy model; LIG, Last interglacial period; LGM, Last glacial maximum period; Bio6, Min Temperature of the Coldest Month; Bio15, Precipitation Seasonality (coefficient of variation); Bio19, Precipitation of the Coldest Quarter; SSP126, Shared socioeconomic pathway 126; this scenario with 2.6 W/m^2^ by the year 2100 is a remake of the optimistic scenario RCP2.6 and was designed with the aim of simulating a development that is compatible with the 2 °C target; SSP370, Shared socioeconomic pathway 370. As an update to scenario RCP4.5, SSP245 with an additional radiative forcing of 4.5 W/m^2^ by the year 2100 represents the medium pathway of future greenhouse gas emissions. This scenario assumes that climate protection measures are being taken; SSP585, shared socioeconomic pathway 585; with an additional radiative forcing of 8.5 W/m^2^ by the year 2100, this scenario represents the upper boundary of the range of scenarios described in the literature. RCP 6.0, representative Concentration Pathway 6.0.
